# Adaptive Information Sharing with Ontological Relevance Computation for Decentralized Self-Organization Systems

**DOI:** 10.3390/e23030342

**Published:** 2021-03-14

**Authors:** Wei Liu, Weizhi Ran, Sulemana Nantogma, Yang Xu

**Affiliations:** School of Computer Science and Engineering, University of Electronic Science and Technology of China, Chengdu 611731, China; 201812081216@std.uestc.edu.cn (W.L.); 202012081713@std.uestc.edu.cn (W.R.); 201814080012@std.uestc.edu.cn (S.N.)

**Keywords:** self-organizing systems, decision-making, information sharing, information relevance, ontology

## Abstract

Decentralization is a peculiar characteristic of self-organizing systems such as swarm intelligence systems, which function as complex collective responsive systems without central control and operates based on contextual local coordination among relatively simple individual systems. The decentralized particularity of self-organizing systems lies in their capacity to spontaneously respond to accommodate environmental changes in a cooperative manner without external control. However, if members cannot obtain observations of the state of the whole team and environment, they have to share their knowledge and policies with each other through communication in order to adapt to the environment appropriately. In this paper, we propose an information sharing mechanism as an independent decision phase to improve individual members’ joint adaption to the world to fulfill an optimal self-organization in general. We design the information sharing decision analogous to human information sharing mechanisms. In this case, information can be shared among individual members by evaluating the semantic relationship of information based on ontology graph and their local knowledge. That is, if individual member collects more relevant information, the information will be used to update its local knowledge and improve sharing relevant information by measuring the ontological relevance. This will enable more related information to be acquired so that their models will be reinforced for more precise information sharing. Our simulations and experimental results show that this design can share information efficiently to achieve optimal adaptive self-organizing systems.

## 1. Introduction

Self-organization is an incarnating characteristic of self organization systems such as swarm intelligence systems, which functions as a complex collective response without central control and operates based on contextual local coordination among relatively simple individual members. The distinctiveness of self-organized systems lies in their capacity to spontaneously form a new team-based organization without external control to accommodate environmental changes. The main concept and self-organization, the fundamental notion of self-organization mechanisms, and the problem of assessing and characterizing such mechanisms are all well defined and presented by [1].

With the advances of self-organization systems research, a representative form which reflects structural dynamics of self-organizing systems, has been widely studied from different perspectives [2] and applied in real applications by building large-scale light-weighted robots in domains such as emergency responses [3], planet explorations [4], and military operations [5]. In those systems, a member simply has a partial observation to the system and the environment [6,7], however, require closely cooperating with each other for achieving a joint, yet complex adaption to the world in a decentralized way by making decisions themselves. Because of the limited knowledge and partial observation to the system, information sharing is imperative for their effective collaboration and interaction. For instance, if an ant searches for and finds a huge piece of food, it must notify the team to find an appropriate member to help carry the food.

Self-organization has been widely studied as an attractive approach for realizing adaptability, robustness, and scalability of large-scale complex systems [8]. For instance, controlling large-scale self-organized networks is presented in [9,10], and Ref. [11] presents a novel distributed algorithm for multiple unmanned aerial vehicles (UAVs) for a search-attack mission self-organization problem. In an attempt to support military operations, Ref. [12] applies concepts in self-organization as a supporting paradigm for UAV Relay Networks. In [13], a model of Self-organizing System of Autonomous Agents is presented. Agents in this model distribute available resources among cells and accumulate energy. Authors in [14] present the self-organisation framework based on coupled dynamical systems and the multivariate information information-theoretic approach. In addition, Ref. [15] applies an effective leadership model (ELM) of the collective decision-making of animal groups to enable self-organizing control mechanisms to adapt to information uncertainty, while authors in [16] develop an ontology model to facilitate knowledge-sharing in complex multi-agent systems.

Despite the many research gains to realize self-organizing systems, effective information sharing in decentralized self-organizing systems still remains a challenge. Some existing works tackle communication decision problems in self-organizing systems using decision theoretical models. With the decision theoretical models, the expected utility of communication actions is leveraged in making communication decisions [17,18,19]. This approach is only suitable for coordination where complete team knowledge is possible. For coordination in large decentralized teams, however, individual members have only local views of both the environment and other member states, which makes computation of the expected utility NEXP-complete [20]. Clearly, obtaining complete team knowledge in large decentralized teams using information sharing strategies is challenging with decision theoretical models. That not withstanding, some attempts have already been made to address this challenge using heuristic algorithms. For example, state factorization of individual members’ observation has been applied in a heuristic information sharing algorithm [21]. This approach maintains a good knowledge base of individual members of the entire system, and carefully determines the relevance of any information that has to be shared. However, there remains a gap on the appropriateness of action selection by members using their local observation, which is limited in scope of both themselves and the environment. What is considered a way forward in this paper is to consider the information sharing process by the individual members as a partial Markov decision process. Principles and objectives of information sharing in large self-organizing teams are explored, providing insight into a practical approach for efficient information sharing.

For a practical approach, instead of indiscriminate information dissemination by individual members to ensure widespread of information in a team, information sharing should target those it will benefit [22], and ultimately to the benefit of the entire team. Therefore, information sharing should be directed, targeting the potential beneficiaries. In addition, in self-organizing systems organization, information about events, capabilities and knowledge about other individual members has inherent relations with one another, and can be leveraged for efficient coordination. For instance, in a combat scenario involving scouter and attacker UAVs, the information that is relayed by a scouter on the location of an enemy is related to the state and locale of the attacker UAVs, and the availability of ammunition. This feature can be used by individuals to predict the information needs of other individuals of the team using the information relevance obtained from their domain knowledge. This particular strategy is novel, and has not been reported in most related works of self-organizing systems’ coordination.

By extending a theoretical decision model into a scalable information sharing problem, we analyze the information utilities between related information. The fundamental point is to prove that information relevance is the intrinsic knowledge relevant to dynamic attributes of the self-organizing system [23]. Next, to compute the relevance of information, we propose a semantic model based on semantic graph. The semantic graph is built with the domain knowledge and information that individual members share. In this context, information refers to semantic information represented by human language but not a communication package that relies on network protocols. In addition, offline relevance computation algorithm is modified to provide a practical online algorithm. In this online computation, the ontology graphs are dynamically updated with the domain knowledge of individual members. Hence, as members continue to receive relevant information, they are able to enrich their knowledge-base and also extend their ontology graphs for improved measurements of information relevance. By doing so, the decision model of individual members is refined, and they are able to get more related information for their domain actions. Finally, we extend the information relevance model to build simulations on sharing coordination. Our experimental results show the feasibility and efficiency of our algorithm in large teams.

The rest of this paper is organized as follows; In Section 2, the decentralized information sharing problem and the need for practical information sharing mechanisms are presented. In Section 3, our ontological information relevance approach is presented in detail. Having presented our approach, experiments and results in a search and rescue task are presented in Section 4. Finally, we present our concluding remarks by summarizing our contributions and future work.

## 2. Decentralized Information Sharing Problem

**Figure 1:**
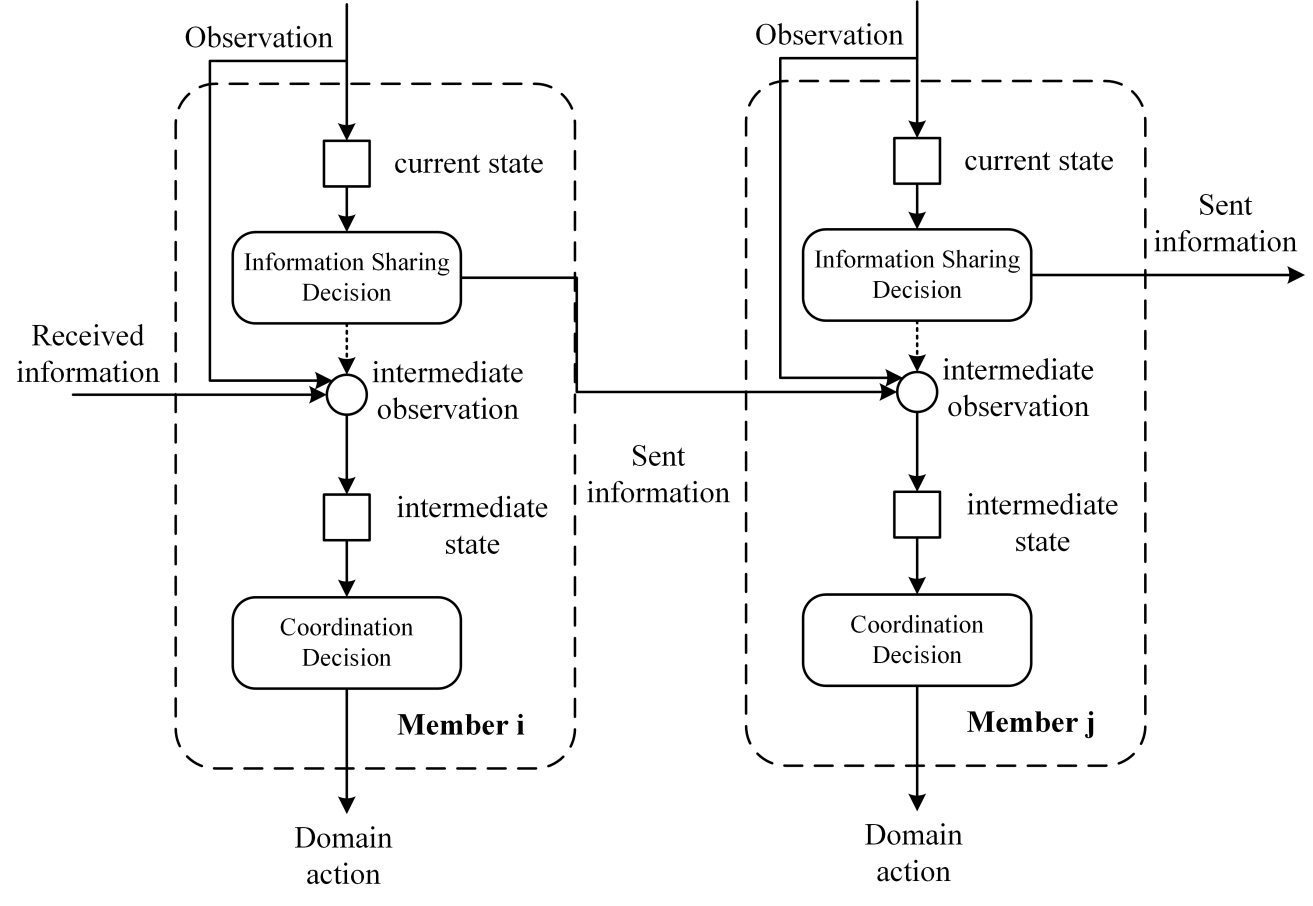
Coordination of the self-organizing system with the information sharing stage.

In this section, we present the nature of decentralized coordination existing between the members of a given self-organized system. The general information sharing model for decentralized members of a team can be explained using the causal processing model shown in Figure 1. Members’ decisions can be explicitly described in two stages. First, members make decisions on how to communicate with some teammates based on their perspective to the environment, so that they can jointly share knowledge. We call this stage information sharing stage. In the next stage termed the domain decision stage, members’ decisions rely on the intermediate observation, subject to the local communication decision, observation, and information received by a member.

### 2.1. Information Sharing Decision Process

At the information sharing stage, decentralized member *a* take information sharing action $CA_{I}$ and changes the information state of team $S_{I}$, while, in the coordination stage, member *a* takes coordination action $CA_{D}$ and changes the domain state of team $S_{D}$. Since the decision process of a member includes these two stages, the team state *S* includes the state of information sharing and domain coordination state: $S:S_{I}\times S_{D}$, and the transition probability function can be computed as shown in Equation (1):

(1) $$\begin{array}{c} P_{D+I}\left(S,S^{\prime }\right)=Prob\left(S_{D}^{\prime },S_{I}^{\prime }|S_{D},S_{I},CA_{D},CA_{I}\right) \end{array}$$

For member *a*, the coordination decision is only based on its intermediate local knowledge, which is fully observable. According to the properties of Bayesian Networks [24], the decision of information sharing and coordination are d-separated and mutually independent when the intermediate observation is given. The whole transition probability can then be written using Equation (2):

(2) $$\begin{array}{c} P_{D+I}\left(S,S^{\prime }\right)=Prob\left(S_{D}^{\prime }|S_{D},CA_{D}\right)\cdot Prob\left(S_{I}^{\prime }|S_{I},CA_{I}\right) \end{array}$$

As an independent decision process, when the information sharing process forwards enough information to support a single member domain decision, we assume it will perform rationally to carry out an action with expected reward. Therefore, $CA_{D}\to R$ and $Prob(S_{D}^{\prime }|S_{D},CA_{D})=1$. The transition probability function for information sharing with the assumption of members’ rational action based on received information can be modeled using Equation (3):

(3) $$\begin{array}{c} P_{D+I}\left(S,S^{\prime }\right)=Prob\left(S_{I}^{\prime }|S_{I},CA_{I}\right) \end{array}$$

Moreover, the reward function also includes these two parts $R:R_{D}\times R_{I}$. The instant reward of an information sharing process is negative $R_{I}\in R^{-}$ because only communication cost occurs. However, a team can benefit when an individual member gets enough related information for a rational joint team decision to be made. For example, if a piece of information denoting a hostile target is approaching and another piece of information talking about obstacle on a particular path reach a single member, the team can make an alternative routing decision so that the team coordination can be improved. Therefore, the information distribution of the team directly decides the domain action when it is assumed that rational actions are expected to be carried out whenever a member gains enough information for their domain decision support: $S_{I}\to CA_{D}$. Since there is $CA_{D}\to R$, the reward function is traceable to information distribution, and the value of immediate reward depends on the corresponding information individual members get.

The information sharing utility function can be defined based on the reference reward that if a set of related information $g_{i}=\left\{I_{i1},I_{i2},\cdots ,I_{ik}\right\}$ is received by one member, a rational joint activity can be carried out in the team toward a reward $R(g_{i})$. To make rational decisions, members of a self-organizing team must make the best trade-off between sharing information for team reward and minimizing communication cost [25].

### 2.2. Decision Theoretical Model

In a large self-organization system, effective information sharing improves each decentralized member’s cooperation and joint actions toward improving their common objective and therefore gaining a higher overall reward. In theory, the information sharing decision process of each member can be also modeled as a partially-observable Markov decision process (POMDP). In order to make rational information sharing decisions, members have to make the best trade-off between sharing information to obtain overall system reward but minimize the communication cost in the sharing process. We adapt the classic definition of information sharing decision model, COMmunicative Multiagent Team Decision Problem (COM-MTDP) model [18], and extend the model as a tuple, $<S,O,\hat{\Upsilon },B,P,CA,ℜ>$, where:

- S denotes the information state of the team. At time *t*, $S\left(t\right)=\underset{a\in A}{\cup }S_{a}\left(t\right)$ where $S_{a}\left(t\right)$ is a set of information states about the system that a member of the team possesses.
- O defines a member’s observation of the environment.
- $\hat{\Upsilon }$ defines the members’ communication sets, which consists of each member’s information, Υ such that $\hat{\Upsilon }\left(t\right)=\underset{a\in A}{\cup }{\hat{\Upsilon }}_{a}\left(t\right)$. Information in member *a*’s communication set ${\hat{\Upsilon }}_{a}\left(t\right)$ is either received or sensed by *a*.
- *B* is the member’s perceived belief state of the team. The probability $B_{a}\left(S\left(t\right)\right)$ is the inferred belief of member *a* of the state of the team S(t) at time *t*.
- $P:B\times \hat{\Upsilon }\times B\to \left[0,1\right]$ is the transition function, which maps the impact of team members’ communication action to the belief state. More formally, at time *t*, $P\left(b,\pi ,b^{\prime }\right)=Pr\left(B\left(t+1\right)=b^{\prime }|B\left(t\right)=b,CA\left(t\right)=\pi \right)$.
- CA represents members’ communication action. CA(a,Υ) is the decision of member *a* on which a neighbor should receive information Υ.
- $ℜ:S\times \hat{\Upsilon }\to R$ is the reward function that returns the cost of choices made by individual members. The rewards obtained over time by individual members adopting a specific course of communication action can be viewed as random variables ${ℜ}_{t}$. For instance, when the individual members carry out their preferred actions based on a set of related information $\Upsilon =\{\Upsilon_{i1},\Upsilon_{i2},\cdots ,\Upsilon_{ik}\}$ received by one of its members, $z_{i}$, the cost of these actions is the reward, $ℜ(z_{i})$, returned by the reward function. A team receives negative $com_{cost}$ values as the cost of information sharing.

When *a* senses or receives a message from someone else, it will update its belief state $B_{a}\left(t\right)$ and communication set, and decides whether the information in ${\hat{\Upsilon }}_{a}\left(t\right)$ should be sent according to its information sharing decision policy. The objective of *a*’s decisions is to send a piece of information so as to reach a state that the whole system could benefit, i.e., a specific member obtains a piece of information it could make a good use of. To measure the expected utility of the member *a*’s decision on where to share the information, we define the expected utility function on $B_{a}\left(t\right)=b_{a}$ under information sharing policy π as shown in Equation (4):

(4) $$\begin{array}{c} Exp^{\pi }\left(b_{a}\right)=\sum_{t=0}^{\infty }P\left(b_{a},\hat{\Upsilon_{a}},b_{a}^{\prime }\right)\times \left(\gamma^{t}\rho \left(b_{a}\right)-com_{cost}\right) \end{array}$$

where $b_{a}^{\prime }$ represents a’s belief state in the next step $B_{a}\left(t+1\right)$ and γ∈(0,1] is the discounted future reward.

Hence, given a belief state of *b*, the reward can be calculated as

(5) $$\begin{array}{c} \rho \left(b\right)=\sum_{S}B\left(S\left(t\right)\right)ℜ\left(S\left(t\right)\right) \end{array}$$

The optimal policy for sharing information in $\hat{\Upsilon }$ is shown in Equation (6), and the expected utility based on value iteration based on Equation (7)

(6) $$\begin{array}{c} \pi^{*}\left(b\right)=argmax_{CA(a,\hat{\Upsilon })}Exp^{*}\left(b^{\prime }\right) \end{array}$$

(7) $$\begin{array}{c} Exp^{*}\left(b\right)=argmax_{CA(a,\hat{\Upsilon })}\left[P\left(b,\hat{\Upsilon },b^{\prime }\right)\cdot \left(\rho \left(b\right)-com_{cost}+\gamma Exp^{*}\left(b^{\prime }\right)\right)\right] \end{array}$$

However, although we can theoretically model the information sharing, this problem in nature is still classified as a DEC-POMDP [26], which has been proven to be NEXP-complete [18]. Therefore, for the intrinsic computation complexity of the problem, the self-organization system has to find a practical way for information sharing by a nature-inspired heuristic idea.

### 2.3. Practical Information Sharing Model

**Figure 2:**
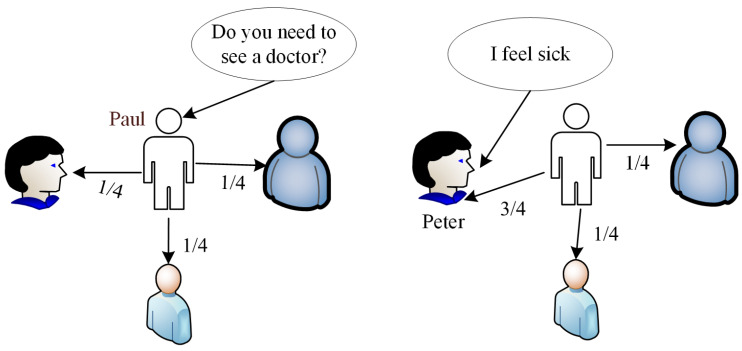
A scenario human information sharing decision concept.

Considering the computational complexity of the theoretical information sharing decision model, we try to find clues for designing efficient information sharing from a nature inspired self-organizing system. Naturally, humans, both biologically and as a society, provides several illustrative examples of such self-organizations [27]. In the human society, information is also communicated and shared in a decentralized way. However, instead of precisely computing its information utility, human beings evaluate and share information in an inferred probability way. We illustrate this information sharing process using Figure 2 to provide a simplistic and easy to comprehend scenario. Assuming, in the midst of his friends, that Paul receives a call “Do you need to see the doctor?”, which suggests that one of his friends should come and see the doctor. However, none of Paul’s friends has indicated to him that he is sick. Thus, Paul decides to share the message “ Do you need to see the doctor?” among his friends. However, if, prior to the call, Paul knew about the health status of his friends i.e., “I feel sick” from Peter, he would prefer to send this message to Peter.

However, this preference does not always lead to a desirable outcome, and the relevance of it demonstrates the effectiveness. Motivated by this information sharing process of humans, we build a practical model for distributed self-organizing systems. In this model, we take advantage of the members’ local knowledge. If we refer to Paul as member *a*, Peter as member *b*, and represent the local knowledge of Paul as $\lambda_{a}$, $\varpi_{a}$ is used to model Paul’s belief state of potential information receivers as compared to the use of *B* in the decision theoretical model. We note that members’ local knowledge solely depends on their previously received information. Similar to human beings, the decision process to match an information sharing action in our practical model is defined in Equation (8):

(8) $$\begin{array}{c} P:\Lambda \times \hat{\Upsilon }\to \left[0,1\right] \end{array}$$

where Λ means sending information, and Paul’s preference to send a piece of information according to its knowledge-base is built as a probability model *P*. According to Equation (8), we build the probability model to denote individuals’ preferred information activity. The model of member *a* is a matrix $P_{a}$, where $P_{a}\left[\upsilon ,b\right]$ represents the preference that information υ is to be sent to an associate *b*(Peter).

When Paul does not have any prior knowledge of his associates’ or his friends’ information needs, any of his friends can become a potential receiver, i.e., Paul will relay the information he receives without any preference. However, following the example in Figure 2, if Paul already knew that Peter was not feeling well (“I feel sick” from Peter), the preference of sending “Do you need to see the doctor?” to Peter becomes higher. Analyzing the preceding scenario, there is a tightly coupled relevance between these two pieces of information i.e., irrespective of the originator of “I feel sick,” there is an increasing preference of sending “Do you need to see the doctor?” since sickness has an intrinsic relationship with seeing a doctor. Therefore, in this practical model, given two pieces of information $q,r\in \hat{\Upsilon }$, the Relevance of *q* and *r* can be computed as the fitness Fit(q,r) between *q* and *r* whose value can be derived from the domain knowledge as discussed in Section 3. This data structure is potentially critical to practical information sharing.

From Equation (8), members in a self-organizing team can rely on their understanding of teammates’ local knowledge to make an informed decision on whether to send its own information to others, as well as update its preference model when the relevance between pieces of information is known. When a new piece of information *q* is received, the practical decision model $P_{a}$ is updated based on the relevance between *q* and any of the information *r* in $\lambda_{a}$. However, when $\lambda_{a}=\left\{\phi \right\}$ i.e., member *a* has no prior knowledge about its neighbors’ local knowledge state, then an unbiased decision process is adopted by *a* for sharing the received information since $P_{a}$ will be equal. When $\lambda_{a}=r$ where *r* is a piece of information sent from associate *b* to *a*, the probability updating function can be written as:

(9) $$\begin{array}{c} P_{a}^{\prime }\left[q,b\right]=P_{a}\left[q,b\right]\times Fit\left(i,r\right) \end{array}$$

This function shows the direct relation of *q* and *r* to *a*’s preference of passing *q* to *b* in order for the team to gain higher expected utility. The update of the preference model of sending *q* by *a* is a recursive process since all other information in $\Lambda_{a}$ is considered. The work in [28] shows the complete updating function:

$$\begin{array}{cc} \forall q,r\in \Upsilon ,b\in N\left(a\right)\;\;\delta_{P}^{\Upsilon }\left(P_{a}\left[q,b\right],m=<r,path>\right)\;\;\;\;\;\;\;\;\;\;\;\;\;\;\;\; & \\ =\left\{\begin{array}{cc} P_{a}\left[q,b\right]\times Fit\left(q,r\right)\times \frac{2}{\left|N\right|} & \mathrm{if}\;q\neq r,b=sender(r)\\ P_{a}\left[q,b\right]\times \frac{1}{\left|N\right|} & \mathrm{if}\;q\neq r,b\neq sender(r)\\ \varepsilon & \mathrm{if}\;q=r,b=sender(q) \end{array}\right. \end{array}$$

It is worth noting that the normalization of *P* is necessary to achieve a common scale. This model has been proven to be feasible when the relevance between information pieces is known [28] based on the practical decision model. However, research on practical approaches to define information relevance is still ongoing [29,30].

## 3. Ontological Approach for Computing Information Relevance

Ontology can generally be viewed as a data model used to represent concepts in a given domain and the relationships between them. It is practical for concepts/objects and their semantic relationships to be represented as ontology graphs based on members’ domain knowledge and shared information. This ontology graph can then be used to compute the relevance between pieces of information. In this section, we introduce an extended method of computing the relevance Fit between pieces of information based on ontology and members’ local dynamic knowledge. To do this, we first discuss with an example what a semantic ontology graph and then goes ahead to present the information relevance model. Next, with an example, we demonstrated how to compute the relevance between concepts and finally present our approach to dynamically update members’ local knowledge and compute the information relevance.

### 3.1. Ontology Graph Representation

Ontologies can be seen as a special kind of graph describing entities, their properties, and relationships between them in a given domain. Ontologies can be used to reason about the objects and their attributes within a domain. Given that self-organizing systems operate in environments where pieces of information and knowledge of individuals can be concretized to extract atomic concepts, correlated with each other by semantic contents [31], this data model can be leveraged to compute relevance between information. To conceptually abstract pieces of information, this paper uses the representation of triples as in Resource Description Framework (RDF).

**Figure 3:**
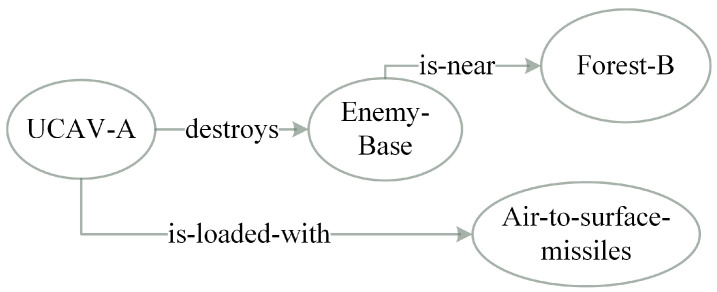
Ontology graph representation of information *“UCAV A loaded with air-to-surface missiles destroyed Enemy Base near forest B”*.

RDF is a standard model for data interchange on the Web. In the RDF data model, resources are represented in the form subject-predicate-object. Similar to web resources, information semantically consists of subjects, predicates, and objects. We refer to subjects and objects, which represent the class or instances of the information source as concepts, and the predicate referred to as property, which describes the attributions of concepts and their relationships.With this approach, a piece of information Υ, consists of concepts $v_{i}$, and properties $p_{j}$, i.e., $\Upsilon =\left\{v_{1},v_{2},\cdots v_{n};p_{1},p_{2},\cdots ,p_{m}\right\}\left(n,m\in R\right)$. For instance, in a combat mission, the information *“UCAV A loaded with air-to-surface missiles destroyed Enemy Base near forest B”* sent by one of the robots can be divided into the semantic set *{“UCAV A”, “EnemyBase”, “Forest B”, “missiles”; “destroyed”, “is-near”, “is-loaded-with”}*. To investigate the relationships between different pieces of information, information in this work is represented with ontology graph as has been in the literature. In an ontology graph G=<V,E>, *V* is used to define the node set that represents the concepts in the semantic ontology, and *E*, the edge set, denoting properties of a piece of information. Figure 3 shows an example of ontology graph of Υ.

Received information in some situations may include multiple communication instructions consisting of multiple clauses. In this case, the multiple communication primitives can be decomposed into single clauses, and its effects will be the summation of all clauses [32]. Thus, a single clause, $clause_{j}=\left\{cl_{j1},cl_{j2},p^{j}\right\}$. For us to be able to perform relevance computations, the defined ontology should be devoid of cyclic or circle definitions. In other words, ontological representation of domain information must use primitive atomic concepts of the domain from which the proposed concepts are created.

### 3.2. Information Semantic Relevance Model

Relying on the intrinsic meaning of words that convey a piece of information, the semantic relationship between pieces of information can be measured [31]. In the model defined above, the semantic components of an information is represented by concepts and their properties. Hence, the relevance between two pieces of information can be measured using the relationship between their concepts and properties. More generally, concepts and properties of pieces of information can reflect the semantic relation between information. Based on the relevance between each concept pairs, the information relevance is modeled without taking into consideration the influence on the relations by properties. Consequently, given two pieces of information, $\Upsilon_{x}$ and $\Upsilon_{y}$, the relevance between them can be defined as Equation (10)

(10) $$\begin{array}{c} Fit\left(\Upsilon_{x},\Upsilon_{y}\right)=\frac{1}{|\Psi_{x}\left|\cdot \right|\Psi_{y}|}\sum_{v_{i}\in \Psi_{x}}\sum_{v_{j}\in \Psi_{y}}Fit\left(v_{i},v_{j}\right) \end{array}$$

where $\Psi_{x}=\Upsilon_{x}-\left(\Upsilon_{x}\cap \Upsilon_{y}\right)$ represents the set of concepts in $\Upsilon_{x}$ but does not belong to the semantic intersection set of $\Upsilon_{x}$ and $\Upsilon_{y}$. Similarly, $\Psi_{y}=\Upsilon_{y}-\left(\Upsilon_{x}\cap \Upsilon_{y}\right)$. The relevance of two pieces of information only takes the relationship of different concepts of them because relevance of the same concepts or word string is 1, and the semantic difference is only caused by the different parts. To compute the average relevance between all different concept pairs of $\Upsilon_{x}$ and $\Upsilon_{y}$, the sum of relevance between all the pairs is divided by the count of different concept groups, i.e., $|\Psi_{x}\left|\cdot \right|\Psi_{y}|$. The similarity of the information $\Upsilon_{x}$ itself is an empty set, i.e., $\Psi_{x}=\emptyset$ and $Fit\left(\Upsilon_{x},\Upsilon_{x}\right)=Fit\left(\emptyset ,\emptyset \right)=1$. $Fit(v_{i},v_{j})$ is the degree of relationship between concepts as presented in Section 3.3.

### 3.3. Concepts Relevance Computation Approach

**Figure 4:**
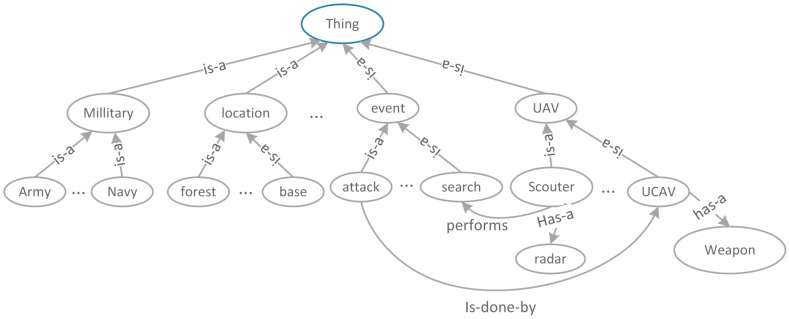
A minimal example initial ontology graph in combat missions.

In the literature, there have been some approaches adopted for computing the similarities between concepts such as one found in Wu et al. [33]. In this work, the relevance between concepts are computed based on the model found in Wu et al. [33]. Given a domain *D*, an initial ontology graph $G_{D}=<V,E>$ is formed in accordance with the knowledge extracted from the domain. Based on the terminologies associated with the domain, the nodes in the tree are expanded beginning with the root node “Thing”—for instance, assuming in a military operation that unmanned aerial vehicles consisting of scouters and combat vehicles (UCAV) are deployed in a forest to destroy enemy bases. In this scenario, the scouters are to search for the bases and communicate to the UCAV to come and destroy the targets. The common knowledge for the members of a self-organizing team can be described as shown in Figure 4.

The members’ decisions are based on the rigidity and non-volatile nature of the domain knowledge and the ontology graph [34]. The relationship between concepts’ definitions and the semantic meaning conveyed by pieces of information is reflected in the ontology graph. This means that the semantic relationship between nodes in an ontology graph can be seen by their distance in the graph. The closer the distance between two nodes, the higher the relevance between concepts they represent. The degree of relationship between two concept nodes $v_{i}$ and $v_{j}$ can be calculated as Equation (11) [33]:

(11) $$\begin{array}{c} Fit\left(v_{i},v_{j}\right)=\frac{2\times len(v_{r},v_{k})}{len\left(v_{i},v_{k}\right)+len\left(v_{j},v_{k}\right)+2\times len\left(v_{r},v_{k}\right)} \end{array}$$

where $len(v_{i},v_{j})$ is the shortest path length between concepts $v_{i}$ and $v_{j}$, and $v_{k}$ is the lowest common ancestor of $v_{i}$ and $v_{j}$. For example, in Figure 4, len(forest,base)=len(scouter,UCAV)=2. The assumption is that the root node, $v_{r}$ of the ontology graph has a shortest path of length 1, i.e., len(Thing,Thing)=1. In addition, it can be said that len(Thing,scouter)=len(Thing,forest)=3. It is worth noting, since the lowest common ancestor of a concept $v_{i}$ is itself and the shortest distance is 0, the relevance of $v_{i}$ is 1, $Fit(v_{i},v_{i})=1$.

To avoid incurring high computational complexities, the computation uses distance between concepts rather than their content [34]. Content based computations require traversing of the entire ontology graph to compute the probability of concepts and all its child nodes. Other than solely computing the shortest distance between nodes, this method weights each edge by the global depth of $v_{k}$. From the above example, the relevance between event and Military is 0.5.

### 3.4. Dynamic Update of the Knowledge Graph

During mission execution, team members encounter or receive new information due to new or uncompleted tasks and events occurring in the environment. The concepts of this information can be modeled as instances of the objects in the ontology graph of the domain knowledge, and they can be described as events [31]. These events come in a form of information whose concepts are object instances in the ontology graph of the domain knowledge. The nature of information received may also differ. This means that some classes of information are more likely to be shared than others since some classes of information may contain more concepts as a result of events and their descriptions. For instance, in our example ontology graph above, a new piece of information *“UCAV U loaded with air-to-surface missiles attacked Enemy Base near forest B”* is more likely to be shared as compared to the information *“UCAV is a subclass of object UAV”* because the former can be classified as an incident or event that occurred and is more likely to be correlated with other events with little semantic similarities based on the domain knowledge. Breaking this down further, in the former information, *“ UCAV”* is related to two concepts, i.e., *“air-to-surface missiles”*, which is an instance of the weapon concept and *“forest B”*, which is an instance of location, and may lead to correlation with more concepts with semantic relations.

For instance, an incident or some piece of information may have been related to the forest, and it is more likely that the former piece of information may connect to some concepts in that information. Hence, although semantics of information reflect the intrinsic relationship between information, the type or class of information in practical applications may lead to new relationships because of the dynamic nature of self-organizing teams. There is therefore the need for members to update their concept ontology graphs to reflect any new information received.

**Figure 5:**
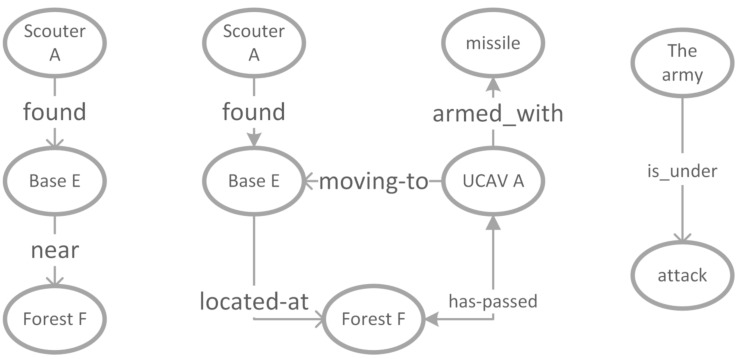
An example of ontology graph with dynamic events in combat missions.

To dynamically update members’ local knowledge, incoming information is dynamically incorporated into the domain ontology graph $G_{D}$, which describes the relationship between concepts. This is done by dividing newly obtained information into concepts and properties according to the factorization presented in Section 3.1 and merged with the former graph to form a new one. For instance, with a new piece of information Υ, the update of $G_{D}$ is done by adding the new concepts in Υ that are not in $G_{D}$ based on property relation, and adding new properties related to concepts that are found in both Υ and $G_{D}$. This means that Υ does not impart any new knowledge on $G_{D}$ if $\Upsilon \in G_{D}$, i.e., all concepts and properties of Υ are in $G_{D}$ including their corresponding edges having the same direction. For example, with an initial knowledge *$\Upsilon_{init}$ = {Scouter A found base E near forest F}* of member of the team, the update of the member knowledge with new pieces of information *($\upsilon_{1}$ = {UCAV U is moving to Base E with missiles}, $\upsilon_{2}$ = {UCAV U has passed forest F}, $\upsilon_{3}$ = {The Army is under attack})* is described in Figure 5.

New nodes are added when information containing new concepts is received, as can be seen in Figure 5. You notice that, after updating the initial graph with $\upsilon_{1}$, all the concepts in information $\upsilon_{2}$ can be found in the resulting graph. Therefore, when new information $\upsilon_{2}$ arrived, the update required adding the connection has-passed between the concepts (Forest F, UCAV A) only. In addition, the piece of information, $\upsilon_{3}$, is irrelevant to the previous information received by the member, and, hence, forms an independent ontology graph. Dynamic information updates may lead to cyclic graphs and more difficulty with avoiding the directed ontology graph as compared to the initial domain knowledge. In the event that there are more relationships between two instances, such as in the following two pieces of information, *the army has procured UCAV* and *UCAV is operated by the army*, a circle is formed between concepts *“UCAV ”* and *“ the army”*. Most of the existing approaches used to compute similarity between concepts are based on a tree, which are not suitable for directed cyclic graphs. In order to solve the cyclic problem, we use the length of the shortest path between two concepts to roughly estimate their relevance.

The relevance between $v_{i}$ and $v_{j}$ is defined as:

(12) $$\begin{array}{c} Fit\left(v_{i},v_{j}\right)=-\gamma log\frac{len(v_{i},v_{j})}{N} \end{array}$$

where $len(v_{i},v_{j})$ is the length of the shortest path between $v_{i}$ and $v_{j}$, γ is a normalization factor, and *N* is the number of concept nodes in the graph. In cases where all vertices connect as a ring, the relevance between vertices pairs can be estimated as the shortest path between any pairs of the vertices. Multiple links between $v_{i}$ and $v_{j}$ reflect the relevance between them. The more links, the more relevant they are to each other. We measure this factor by considering the length of distance between the two concepts to be shorter than 1. For *n* links between $v_{i}$ and $v_{j}$, $len\left(v_{i},v_{j}\right)=\frac{1}{n}$. The direction of arcs are ignored in our calculations.

Since the relevance of information is determined by both the semantic meaning of concepts as contained in the information and the events the concepts describe, the relevance of concepts in information can be modeled as:

(13) $$\begin{array}{c} Fit\left(v_{i},v_{j}\right)=\alpha Fit_{off}\left(v_{i},v_{j}\right)+\left(\left(1-\alpha \right)Fit_{on}\left(v_{i},v_{j}\right)\right) \end{array}$$

where $Fit_{off}\left(v_{i},v_{j}\right)$ and $Fit_{on}\left(v_{i},v_{j}\right)$ are the online and offline relevance computed using Equations (11) and (12), respectively, and α is a normalization factor. $Fit_{off}\left(v_{i},v_{j}\right)$ measures the semantic relevance of the concepts, where concepts of information are regarded as instances of the objects in the initial domain knowledge. Hence, $Fit_{off}\left(v_{i},v_{j}\right)$ is actually the relevance between concepts that $v_{i}$ and $v_{j}$ respectively belong to. In this way, the relevance of information can be computed as shown in Equation (13).

We give an illustration of the computation using Figure 4 and Figure 5b. Suppose we want to compute relevance between *$\upsilon_{1}$=“Scouter A found base E near forest F”* and *$\upsilon_{2}$ = “UCAV U has passed forest F”*, with α=0.5, β=1. Thus, $Fit_{off}\left(E,U\right)=0.25$ and $Fit_{on}\left(E,U\right)=0.85$. This implies that Fit(E,U)=0.55. Hence, $Fit(\upsilon_{1},\upsilon_{2})=0.55$. This is similar for *$\upsilon_{3}$ = “The Army L is under attack”* and $\upsilon_{2}$. $Fit_{off}\left(L,U\right)=0.25$ and $Fit_{on}\left(L,U\right)=0.0$. Therefore, $Fit(\upsilon_{3},\upsilon_{2})=0.125$. From above, it is evident that the information as contained in $\upsilon_{2}$ has concepts that have intrinsic meaning or semantic relevance to the incident of finding a base and a UCAV moving in to destroy it in $\upsilon_{1}$ as it has to $\upsilon_{3}$.

In as much as our proposed model of information relevance computation is applicable in situations where the coordination of decisions and task assignment is shared as members’ interactive information, we note that the design of increasing probability of sending relative information to the previous information sender is not appropriate—for instance, if the piece of information, *which UCAV can attack base E near forest A?*, previously sent from member *b* to *a*, is sent to member *c*. Instinctively, if the information was false, member *a* should be more likely to rather share with *c*, *There is no base near forest A* since it is *c* that should be in good position to reach the member that would be taking up the task to rescind. On the other hand, if *b* sends task assignment information as “I am attacking base E near forest A,” a’s probability of sharing *i* to *c* will be increased as described in Section 2.3. To cater for relevance in shared coordination, our decision model is extended to share information *i* based on a piece of coordination information Δ previously received:

$$P_{a}^{\prime }\left[i,b\right]=P_{a}\left[i,b\right]\times Fit\left(i,\Delta \right)\;\;\;\;where\;\;\;b=receiver\left(\Delta \right)$$

where Fit(i,Δ) is the relevance of *i* and Δ, and member *b* received Δ from *a*. The idea is for related information to be shared with a coordination receiver so that individual members that accepted the assignment of resources or tasks can be rewarded.

## 4. Simulation and Results

To manifest the efficiency of our information sharing design based on ontological relevance computation, we built a self-organizing robot swarm in a search and rescue domain [35,36]. The simulation was designed in the Java programming language based on an eclipse RDF4J framework [37] and JGRAPHT [38] library. By applying our model of information sharing with ontological relevance computation, we hypothesize that this design will improve the decentralized information sharing process between robots and in turn enhance their domain performance in kinds of benchmarks.

### 4.1. Simulation Setup

In this simulation, a group of heterogeneous robots was deployed to start from their base and perform search and rescue tasks. There are two basic types of robots: search robots and rescue robots. Search robots are equipped with a large range of sensors, and can move faster than rescue robots to be able to explore and find victims in undetected areas. To save a detected victim, one or more rescue robots receiving the information are required to go to the distressed zone and perform the rescue task. However, different types of victims require different rescue robots. In our abstract simulation, the heterogeneity is represented as different colors in both victims and rescue robots. For example, the red color of victims can only be saved by red colored rescue robots. In addition, all the robots are equipped with wireless communicators that allow communication only among a particular range of robots for a possible peer-to-peer information sharing. Thus, robots are required to make a good balance of information sharing in the group in order to improve team coordination and safeguard the communication process.

**Figure 6:**
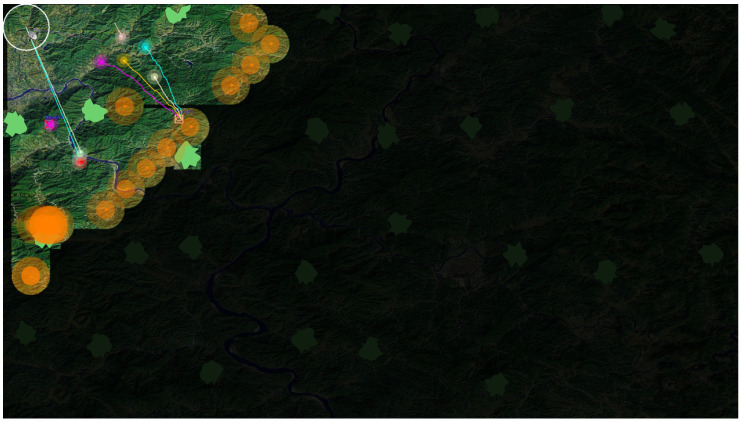
Simulation setup. The opaque area is an area that has not been searched yet. The green shapes represent obstacles, the orange circles depict the radar range of search robots, and the different lines represent path plans of rescue robots while victims are depicted by boxes.

**Figure 7:**
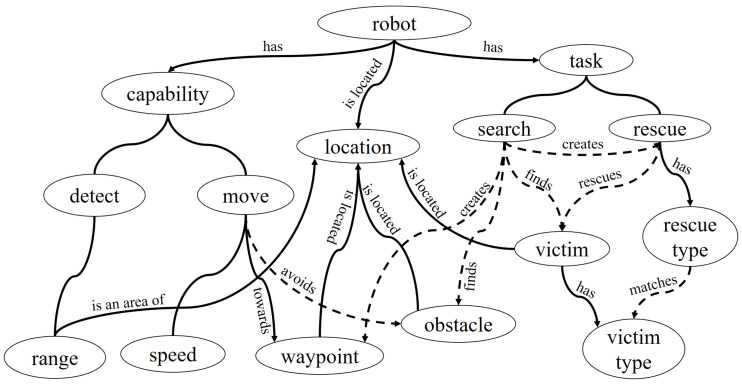
Ontological representation of the simulation.

The simulation starts by dispatching search robots from the base to detect different unexplored areas for obstacles and victims. When a victim is found, the information is shared for a rescue operation to commence. An ideal type of rescue robot, if idle and obtaining this information, activates a rescue mission by moving to the victim’s location. All the robots are encoded with a decentralized path planning algorithm. However, their performance is better if they obtain knowledge of all obstacles in advance other than detecting the same by themselves. When a rescue robot finishes its task and gets no further information on another victim to attend to, there are two options: wandering to find victims by itself or returning to the base, while both options consume their fuel (this process is briefly depicted in Figure 6). Therefore, in the simulation, information sharing is necessary to improve the team performance and can be evaluated by two statistics: amount of information being communicated (the less the better) and the sum of distance that all the robots traveled (the less the better). All the simulation setup can be briefly illustrated as an ontological map in Figure 7. To evaluate our design, we encoded three different algorithms:

- Replan_heuristic_comm: represents our designed ontological relevance based information sharing algorithm where robots inference semantic information received and pro-actively share the information so that less information is relayed, but robots are rewarded with shorter length of routes being traveled for saving the same number of victims.
- Replan_without_comm: Performing search victims and rescuing them by the robots themselves without sharing any information. It is considered as the baseline of team performance.
- Replan_free_comm: each robot connects with all others, which forms a fully connected network. A robot shares its own information to any of the others freely. As there is no information sharing cost being considered, robots are able to get the full map of information. Although its performance could be understood as the upper limit, the information sharing cost should be huge.

**Figure 8:**
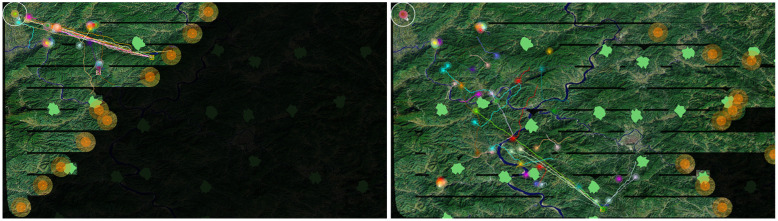
Simulation screen shots. On the left is a few moments after simulation started, when the victims found are rescued by appropriate robots. Towards the end (right), as the search area reduces drastically, some of the search robots started returning to the base while the rescue is ongoing.

In each simulation, unless otherwise explained, the default setting is: there are 10 search robots and 50 rescue robots to rescue the same number of victims randomly placed in the map. Victims are grouped into 10 types in different colors. Rescue robots are attributed with the same number of types and five robots in each type. Moreover, 30 obstacles are set randomly in the environment. Several simulation screen shots are shown in Figure 8.

### 4.2. Simulation Results as Time Varies

**Figure 9:**
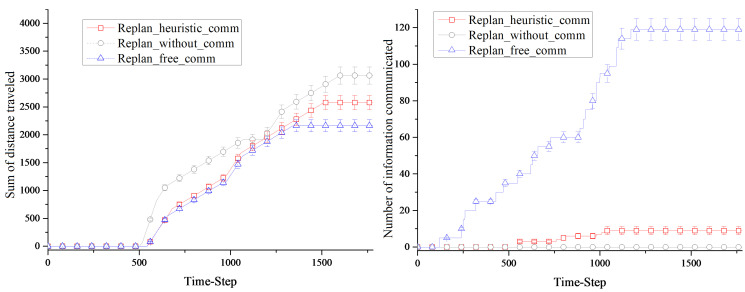
Relevance of robot planning results and time.

The first simulation investigated the algorithm’s performance over time. We use default settings in this simulation, and settings remained unchanged during the process. Sum of distances traveled and number of information communicated were recorded at each time step of the simulation. The results are shown in Figure 9. Use of *Replan_free_comm* achieved the shortest distance traveled with the greatest cost in communication. Without communication, simulation using *Replan_withou_comm* resulted in the largest sum of distance traveled. In fact, robots can only plan paths based on their own knowledge without information sharing. *Replan_heuristic_comm* achieved a moderate sum of distance with a much lower communication cost than the first algorithm. Taking both factors into consideration, our algorithm has the best utility.

As simulation proceeds and more victims are spotted, more rescue robots are assigned with rescue tasks. During this process, more knowledge is discovered and shared causing communication costs to go up. The total messages sent between robots using the *Replan_heuristic_comm* algorithm are always less than *Replan_free_comm* algorithm. Because of the free and all connected communication, robots using *Replan_free_comm* algorithm are always up-to-date, and manage to achieve the lowest total travel length cost with a significant cost in communication. Due to the lack of communication, robots using *Replan_without_comm* are not able to replan their path ahead of time, which leads to the highest travel length cost among three algorithms.

### 4.3. Scalability under Variable Number of Rescue Robots

**Figure 10:**
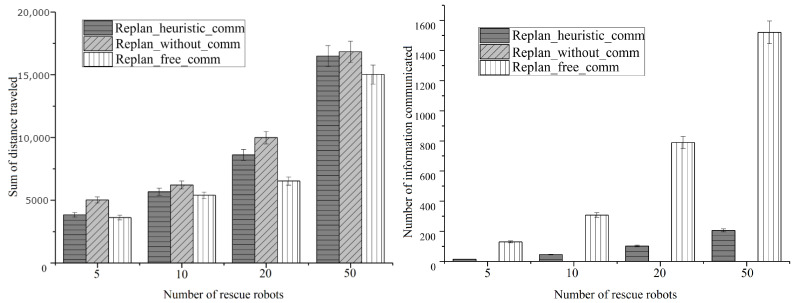
Simulation results under variable team size.

The second simulation examined in more detail the scalability of our algorithm under different team size settings. We set up five scout robots in all settings and 5, 10, 20, and 50 rescue robots and victims for each setting. Performance was measured by the sum of distances traveled by rescue robots and the amount of information communicated between robots. The results are shown in Figure 10. As the results suggest, our algorithm produced less communication costs while maintaining a moderate distance cost. We give the same conclusion as the first simulation. Our simulation keeps the best utility among different team sizes.

### 4.4. Scalability under Variable Exploration Rate

**Figure 11:**
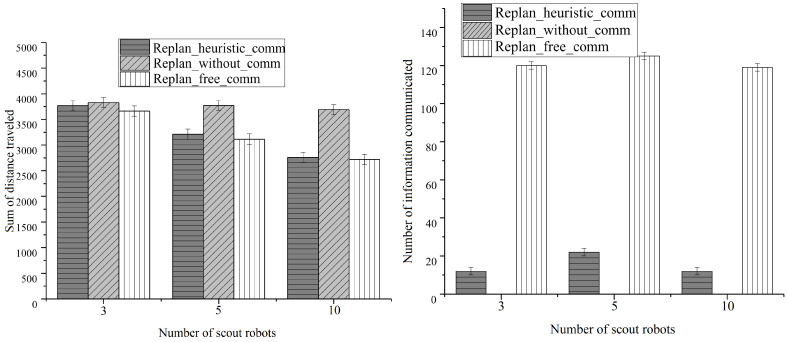
Simulation results under variable exploration rates.

In this simulation, we focus on the scalability under varied exploration rates. We set up five rescue robots, five victims in all settings and 3, 5, and 10 scout robots for each settings. The results are shown in Figure 11. As the results show, our algorithm maintains the best utility among three algorithms under all conditions.

## 5. Summary and Future Works

In this paper, we presented information sharing as a fundamental decision problem, which directly influences the performance of large scalable self-organizing systems. Since computing utility from theoretical decision models is not feasible for large teams, we proposed a heuristic approach that utilizes the relevance of information to aid members in self-organizing teams with making communication decisions. We also defined the numerical representation of the key factors of information relevance in this heuristic approach, and introduced an ontological method to evaluate it. Our experiments proved that this design is feasible for self-organizing teams coordination in many application domains.

Critically, in our lightweight simulation, we only considered a few ontological instances, and abstracted many important factors required for real domain coordination. In the future, we will build ontology databases and encode our algorithm into a large scale team coordination domain such as a city rescue operation, naval combats operations, etc. Secondly, an extension of the information sharing model is imperative because our approach is based on a peer-to-peer information sharing, whereas, in many application domains, the communication infrastructure is based on broadcasting.
